# Serum neurofilament light chain in fibromyalgia: comparative evidence of neuronal injury across chronic pain conditions

**DOI:** 10.1097/PR9.0000000000001423

**Published:** 2026-03-24

**Authors:** Joel Fundaun, Eoin Kelleher, Lydia Coxon, Amanda Wall, Jishi John, Kurtis Garbutt, Andreas C. Themistocleous, Yuki Terajima, David L. Bennett, Katy Vincent, on behalf of the ENDOx/EndoPain and TRIPP study team, Anushka Irani, on behalf of the PAIN-less study team, Annina B. Schmid

**Affiliations:** a Nuffield Department of Clinical Neurosciences, University of Oxford, Oxford, United Kingdom; b Department of Anesthesiology, Perioperative and Pain Medicine, Stanford University School of Medicine, Palo Alto, CA, USA; c Department of Anesthesia, Critical Care and Pain Medicine, Massachusetts General Hospital, Boston, MA, USA; d Nuffield Department of Women's and Reproductive Health, University of Oxford, Oxford, United Kingdom; e Department of Pain Medicine, Aichi Medical University, Aichi, Japan; f Division of Rheumatology, Mayo Clinic Florida, Jacksonville, FL, USA

**Keywords:** fibromyalgia, Small fibre neuropathy, Endometriosis, Neurofilament light chain, Neuropathic pain

## Abstract

**Introduction:**

Evidence increasingly shows small nerve fibre pathology in subsets of people with fibromyalgia through skin biopsy, corneal confocal microscopy, and microneurography. However, blood-based biomarkers of ongoing neuronal damage, particularly neurofilament light chain (NfL), an axonal structural protein, are not well established.

**Objectives:**

The aim of this study was to evaluate serum NfL concentrations in fibromyalgia compared with other chronic pain conditions and assess their relationship with neuropathic-like pain.

**Methods:**

We measured serum NfL using Simoa SR-X NF Light v2 digital-immunoassay in 60 participants with fibromyalgia, 61 with endometriosis (another traditionally considered non-neuropathic pain condition), 24 with small fibre neuropathy (a recognised neuropathic pain condition), and 30 healthy controls. Serum NfL concentrations were compared between the groups controlling for age and body mass index. Neuropathic-like pain was assessed using the painDETECT questionnaire for fibromyalgia and endometriosis and NeuPSIG grading criteria for small fibre neuropathy.

**Results:**

Compared with healthy controls (mean concentration = 6.77 pg/mL, Z-score = −1.15), serum NfL concentrations were significantly elevated in fibromyalgia (mean concentration = 8.52 pg/mL *P* = 0.048; Z-score = −0.30, and *P* = 0.006) and small fibre neuropathy (mean concentration = 8.98 pg/mL, *P* = 0.004; Z-score = 0.23, *P* = 0.0001), with endometriosis representing an intermediate phenotype (mean concentration = 5.57 pg/mL, *P* = 0.44; Z-score = −0.41, *P* = 0.024). Using PainDETECT, 44% of fibromyalgia and 26% of endometriosis participants met likely neuropathic pain criteria; using NeupSIG grading, 96% of small fibre neuropathy participants met probable/definite criteria.

**Conclusion:**

Our study provides serological evidence of nerve pathology in fibromyalgia at comparable levels with small fibre neuropathy, although this did not correlate with neuropathic-like pain presence or severity. These findings highlight the need for subgroup-specific treatment strategies.

## Abstract 2

Supplemental Digital Content is Available in the Text.

Serum neurofilament light chain levels were similarly elevated in fibromyalgia and small fibre neuropathy, suggesting nerve pathology in a subset of individuals with fibromyalgia.

## 1. Introduction

Fibromyalgia is a prevalent chronic pain condition characterized by widespread pain commonly presenting with fatigue, sleep disturbance, and cognitive impairments.^32,46^ The etiology of fibromyalgia is heterogeneous and thought to encompass numerous biopsychosocial factors that vary among individuals.^10,46^ Although pain in fibromyalgia is predominantly associated with a nociplastic phenotype,^33^ increasing evidence demonstrates a subset exhibits neuropathic-like pain and structural abnormalities in small fibre terminals within the skin.^28,50^

The pooled prevalence of small fibre nerve pathology in fibromyalgia is approximately 49%, based on recent meta-analysis of nerve fibre densities from skin biopsies and corneal confocal microscopy.^24^ Aligned with these morphological findings, increased spontaneous activity and abnormal firing patterns in peripheral C-fibre nociceptors have been shown using microneurography in people with fibromyalgia.^48^ Although replicated across many laboratories internationally,^50^ the role that such nerve fibre dysfunction plays in the development or maintenance of fibromyalgia remains unclear, underscoring the need for additional biomarkers of active neuronal injury to better understand these pathophysiological processes.

Blood-based biomarkers have shown utility as a noninvasive alternative for detecting axonal involvement.^36^ One increasingly studied biomarker is neurofilament light chain (NfL), which is a cytoskeletal protein found in axons that is released following injury. Neurofilament light chain has emerged as a consistent blood-based biomarker of active neural injury in diseases affecting both the central^23,37^ and peripheral nervous systems.^21^ A recent preliminary study showed elevated plasma NfL concentrations in participants with fibromyalgia compared with controls. However, these findings were from a small cohort and did not adjust for body mass index (BMI), which significantly alters NfL levels.^45^ Although previous studies demonstrate that reduced cutaneous and corneal small nerve fibre densities did not correlate with pain severity,^28,50^ this relationship has not yet been assessed using NfL.

Similar to fibromyalgia, signs of nerve pathology and questionnaire-based classifications of neuropathic-like pain have been identified in other chronic pain conditions traditionally not considered to be of neuropathic origin.^4,22^ For instance, endometriosis-associated pain is traditionally considered nociceptive^12^; however, 30% to 45% demonstrate sensory loss of function over the abdomen,^13^ and approximately 40% are classified to have neuropathic-like pain according to the DN4 or painDETECT screening tools.^8,14^ However, such questionnaires may not purely capture neuropathic components, as evidence suggests that they can identify signs of central sensitization in patients with neuropathic-like pain.^52^ Neurofilament light chain may address these limitations by objectively identifying axonal injury across chronic pain conditions.

To characterise NfL across a spectrum of nerve injury, this study compared NfL levels in traditionally considered non-neuropathic chronic pain conditions, including fibromyalgia and endometriosis, compared with small fibre neuropathy—a recognised neuropathy and neuropathic pain condition, and pain-free healthy controls. Given these conditions demonstrate varying levels of neuropathic-like pain, a secondary aim was to assess the relationship between NfL levels and neuropathic-like pain across all cohorts.

## 2. Methods

### 2.1. Participants

This study includes participants and blood samples from four separate cohorts, including fibromyalgia, endometriosis, small fibre neuropathy, and healthy controls. Full details of the diagnostic criteria for patient cohorts are included in Supplemental Materials (http://links.lww.com/PR9/A391).

#### 2.1.1. Fibromyalgia

The fibromyalgia cohort included participants from a randomised controlled trial of digital cognitive behavioural therapy for insomnia. Participants who attended the baseline prerandomisation visit between July 2023 and September 2024 and had a serum sample taken were eligible. This trial was nested within a larger prospective cohort study titled “Characterisation of Pain in Patients with Musculoskeletal Disease: A Longitudinal, Observational Study with an Embedded Feasibility Window of Opportunity Sleep Study” (Pain-LESS). Ethical approval was received from South Central—Oxford B Research Ethics Committee (reference: 19/SC/0168). The trial was preregistered with full details on clinicaltrials.gov (reference: NCT05962138).

Participants with fibromyalgia were 18 years or older and met the 2016 American College of Rheumatology diagnostic criteria for fibromyalgia, which requires the presence of generalised pain (at least 4 out of 5 body regions) for at least 3 months alongside nonpain symptoms, such as fatigue, sleep disturbance, and cognitive problems.^60^

#### 2.1.2. Endometriosis

Participants in the endometriosis cohort, serving as a chronic pain control condition traditionally characterised as nociceptive pain, were consented to ENDOX (approved by NHS Research Ethics Committee - South Central Oxford A REC reference 09/H0604/58, n = 62, recruiting between November 2012 and May 2018) and/or a concurrent study EndoPain2 (which recruited between April 2016 and September 2017, n = 24). The ENDOX study collected data from patients undergoing laparoscopy and involved the collection of blood samples and pelvic biospecimens on the day of surgery and the completion of comprehensive questionnaires according to the World Endometriosis Research Foundation Endometriosis Phenome and Biobanking Harmonization Project (WERF EPHect) recommendations.^59^ All participants were older than 18 years and before menopause. The EndoPain2 study received ethical approval from NRES Committee South Central Oxford B (15/SC/0372) and involved brain imaging (fMRI), questionnaires, and biospecimen collection. This study recruited individuals who had previous surgical diagnosis of endometriosis (within the past 5 years) before repeat surgery for persistent or recurrent pelvic pain. All women reported nonmenstrual pelvic pain of an intensity ≥4/10 occurring on at least 7 days per calendar month, for at least 6 months. Women were excluded if they presented with contraindications for magnetic resonance imaging or they had used centrally acting drugs in the past 3 months (eg, antiepileptics, antidepressants, anxiolytics).

#### 2.1.3. Small Fibre Neuropathy:

Participants with idiopathic small fibre neuropathy were included as a positive control with diagnosed neuropathy and consented to the “Pain in Neuropathy Study” (NRES reference: 10/H07056/35, approved by Riverside research ethics committee) and the “Pain in Peripheral Nerve Lesions” study (REC reference 18/SC/0263, approved by South Central—Oxford C Research Ethics Committee) between July 2015 and September 2024. Detailed descriptions of the phenotyping has been previously published.^44^ Participants had clinically confirmed idiopathic small fibre neuropathy, were aged older than 18 years, and not pregnant.

#### 2.1.4. Healthy controls

Thirty healthy control participants were recruited through the Pain in Neuropathy Study (NRES reference: 10/H07056/35) and Pain in Peripheral Nerve Lesion study (PiPL, Oxford C Research Ethics Committee, REC reference 18/SC/0263) from July 2015 to June 2024. Healthy participants were older than 16 years without current pain and the absence of ongoing chronic pain. People with a history of any central or peripheral neuropathy or systemic diseases potentially causing a neuropathy (eg, diabetes) were excluded. Healthy controls were proportionally age and sex matched to individuals in the fibromyalgia cohort, as this was our main group of interest.

### 2.2. Demographic data and neuropathic pain classification

Demographic details, including age, sex, height, weight, were available for each participant. Symptom duration was recorded through self-report apart from the endometriosis cohort, where symptom duration was calculated from the youngest age of pelvic pain, period pain, or pain with sex.

Neuropathic pain for participants with small fibre neuropathy was evaluated using the Neuropathic Pain Grading System from the Neuropathic Pain Special Interest Group (NeuPSIG) of the International Association for the Study of Pain.^19^ This hierarchical grading system classifies the certainty of neuropathic pain using 3 grades: (1) possible requiring a history of relevant neurological lesion or disease and pain within a neuroanatomically plausible distribution; (2) probable requiring sensory signs within the same neuroanatomically plausible distribution; and (3) definite requiring a diagnostic test confirming a somatosensory lesion. The diagnostic test used in this study for definite neuropathic pain was reduced intraepidermal nerve fibre densities compared with age- and gender-matched controls from published normative values,^3^ which was available in 23 of 24 patients with small fibre neuropathy. As the neuropathic pain grading was not available for the fibromyalgia and endometriosis cohorts, we used the painDETECT questionnaire, a validated screening tool for neuropathic pain, ^20^ which previously demonstrated moderate sensitivity, specificity, and accuracy compared with the neuropathic pain grading system in patients with musculoskeletal pain and suspected nerve lesion.^53^ In the endometriosis cohort, painDETECT scores were not always collected at the same time as the blood samples for those in the TRiPP study. The painDETECT classification suggesting neuropathic-like pain used established cutoffs of unlikely: <13, unclear: 13 to 18, and neuropathic-like pain ≥19.^20^

### 2.3. Blood processing

Blood was collected from the cubital fossa for all participants (BD Vacutainer Tube SST Advance). Within 30 to 240 minutes after collection, serum for all cohorts was centrifuged at approximately 2000 to 2500*g* per minute at 4°C for 10 minutes. The serum was collected and stored at −80°C for batch analysis.

### 2.4. Neurofilament light chain protein levels

Neurofilament light chain protein concentrations were analysed using the Simoa SR-X and NF-Light v2 Advantage Kit (Quanterix, Billerica, MA). All serum samples were run in duplicate using a random combination of participants with pain and healthy controls per plate, as well as a separate calibration curve for each plate.

Because there is currently no gold-standard approach for analysing NfL levels, we used 2 complementary methods. First, we calculated absolute serum NfL concentrations (pg/mL) as the mean of duplicate measurements. Second, we used participants' absolute NfL concentrations to calculate age- and BMI-adjusted z-scores using a previously published reference population of 4532 neurologically healthy controls from 4 US and European cohorts spanning 6 age decades.^6^ This control reference database excluded participants with major diseases, including central nervous system disorders. In the original reference population study, age and BMI were identified as the most significant factors independently associated with NfL levels, whereas sex showed no significant association.^6^ Individual NfL z-scores provide a normalised value where z-scores of zero represents the mean score expected for a person of the same age and BMI and demonstrate the number of standard deviations NfL levels differ compared with the previously collected reference population. Adjustments for differences in assay kits between our study and the general population cohort were made according to technical recommendations (Simoa, Quanterix).

### 2.5. Statistical analysis

Because this was a secondary analysis of existing cohorts, no sample size calculation was performed, but rather all chronic pain participants with available blood samples were included. Data were analysed using R software (version 4.4.1). The Kolmogorov–Smirnov test and visual inspection were used to assess data normality. Normally distributed data were presented as mean and standard deviation (SD) and as median (Mdn) and interquartile range for non-normally distributed data. Statistical significance was set at *P* < 0.05.

Absolute NfL concentrations were first compared between the groups using an ANCOVA with age and BMI included as continuous covariates in the model, as both influence NfL concentrations.^6^ Tukey post hoc test was performed to evaluate pairwise group differences. Next, age- and BMI-adjusted NfL z-scores were analysed using ANOVA followed by Tukey post hoc testing. We report estimated pairwise group differences with Tukey-adjusted *P* values and effect sizes as Hedges g with 95% confidence intervals. We did not correct for sex, as it does not seem to influence NfL levels.^6^

To evaluate NfL levels based on neuropathic pain status, participants were classified as having “neuropathic” or “unlikely neuropathic” pain using cohort-specific methods. For fibromyalgia and endometriosis participants, neuropathic-like pain was defined as the more conservative painDETECT cutoff >18, whereas unlikely neuropathic pain was defined as scores ≤18. For small fibre neuropathy participants, neuropathic pain included those graded as having at least probable neuropathic pain. Absolute NfL concentrations were compared between neuropathic pain groups using ANCOVA (controlling for age and BMI), and NfL z-scores were compared using ANOVA. Spearman partial correlations (controlling for age and BMI) were used to evaluate associations between NfL levels and neuropathic pain severity in fibromyalgia, assessed using both total painDETECT scores and individual item scores. The Benjamini–Hochberg method was applied to correct for multiple testing. Individual painDETECT scores by item were not available from the other cohorts.

## 3. Results

### 3.1. Participant characteristics

Participant characteristics are included in Table 1. The median age was 50 years for participants with fibromyalgia, 40 years for small fibre neuropathy, and 45 for healthy controls. As expected, the endometriosis cohort was younger with a median age of 34 years. Most participants in each cohort were female (93.3% [56/60] in fibromyalgia, 100% [61/61] in endometriosis, 50% [12/24] in small fibre neuropathy, and 90% [27/30] in healthy controls). For participants with small fibre neuropathy, 41.7% (10/24) were classified as probable small fibre neuropathy and 58.3% (14/24) as definite.

**Table 1:** Participant characteristics.

	Fibromyalgia (n = 60)	Endometriosis (n = 61)	Small fibre neuropathy (n = 24)	Healthy control (n = 30)
Age (Mdn/IQR)	50.0 (17.3)	34.0 (12.0)	40.5 (17.5)	45.0 (18.0)
Sex (female, %/n)	93.3% (56/60)	100% (61/61)	50% (12/24)	90% (27/30)
BMI (Mdn/IQR)	29.4 (9.2)	25.1 (7.2)	27.5 (4.1)	23.9 (7.6)
PainDETECT total score (mean/SD)	18.3 (6.8)	14.5 (7.8)*	NA	NA
Symptom duration, y (Mdn/IQR)	7.0 (11.0)	15 (12)†	4 (4.5)	NA

* Twenty missing.

† Thirteen missing.

BMI, body mass index; IQR, interquartile range; Mdn, median; SD, standard deviation.

### 3.2. Neuropathic-like pain is present in the majority of participants with fibromyalgia and small fibre neuropathy

**Figure 1.**
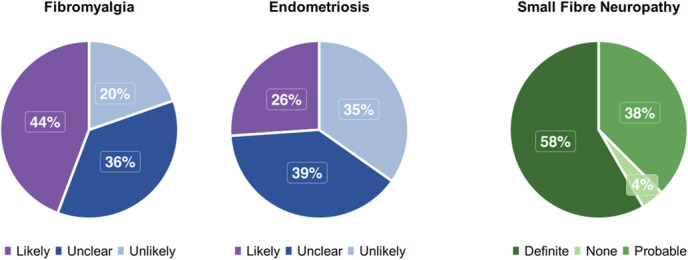
The likelihood of neuropathic pain across chronic pain conditions. Fibromyalgia and endometriosis values were derived using painDETECT cutoff scores; the NeuPSIG grading criteria were used in the small fibre neuropathy cohort.

Mean painDETECT scores were 18.3 (SD 6.8) for fibromyalgia and 14.5 (SD 7.8) for endometriosis (Table 1). The proportion of participants classified as having neuropathic-like pain using the painDETECT questionnaire was 44% (27/61) in fibromyalgia and 26% (12/46) in endometriosis (Fig. 1). Neuropathic pain as classified using the NeuPSIG grading system in small fibre neuropathy demonstrated that 58% (14/24) had definite neuropathic pain and 38% (9/24) had probable. None had possible neuropathic pain and 1 participant reported numbness without pain and was classified as having no neuropathic pain.

### 3.3. Elevated concentrations of neurofilament light chain in fibromyalgia and small fibre neuropathy

**Figure 2.**
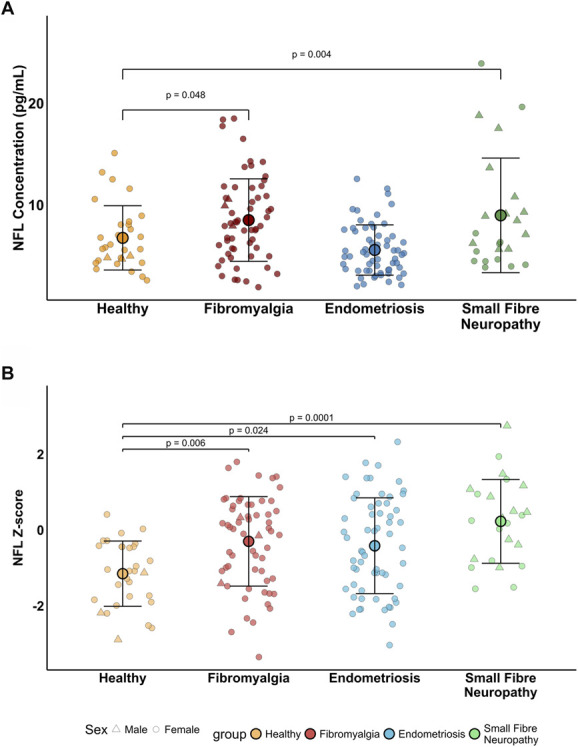
Serum NfL concentrations and z-scores across chronic pain conditions. (A) Absolute NfL concentrations (pg/mL) compared between the groups using ANCOVA adjusted for age and BMI. (B) Age- and BMI-adjusted NfL z-scores compared between the groups using ANOVA. Number of participants with NfL data per group: Healthy = 30; fibromyalgia = 60; small fibre neuropathy = 33; endometriosis = 61.

Serum absolute NfL concentrations were different between the groups (F[3,169] = 28.28, *P* < 0.001). Post hoc testing revealed elevated NfL concentrations in participants with fibromyalgia (mean concentration = 8.52 pg/mL, *P* = 0.048) and small fibre neuropathy (mean concentration = 8.98 pg/mL, *P* = 0.004) but not in endometriosis (mean concentration = 5.57 pg/mL, *P* = 0.43) compared with healthy control participants (mean concentration = 6.77 pg/mL, Fig. 2A, Supplemental Table 1, http://links.lww.com/PR9/A391). There were no significant differences in absolute NfL concentrations comparing any other groups (*P* > 0.08, Supplemental Table 2, http://links.lww.com/PR9/A391).

Age- and BMI adjusted NfL z-scores also showed group differences (F[3,171] = 7.56, *P* < 0.001). Post hoc testing revealed significantly higher NfL z-scores in participants with fibromyalgia (*P* = 0.006), small fibre neuropathy (*P* = 0.0001), and endometriosis (*P* = 0.024) compared with healthy controls (Fig. 2B, Supplemental Tables 1, 3, http://links.lww.com/PR9/A391). Between-group adjusted mean differences and effect size estimates are detailed in Supplemental Tables 4 and 5, http://links.lww.com/PR9/A391.

### 3.4. Neurofilament light chain levels were not related to neuropathic-like pain

**Figure 3.**
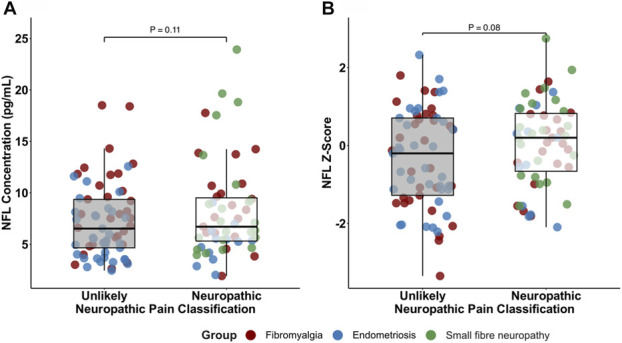
Serum NfL levels were not different based on neuropathic pain state. (A) Serum NfL concentrations (pg/mL). Results from ANCOVA controlling for age and BMI. (B) Age- and BMI-adjusted NfL z-scores. Results from ANOVA. Neuropathic pain defined as painDETECT scores ≥19 (fibromyalgia, endometriosis) or probable/definite neuropathic pain using the NeuPSIG criteria (small fibre neuropathy). NFL, neurofilament light chain.

There were no significant differences in serum NfL levels in participants dichotomized into neuropathic and unlikely neuropathic pain across cohorts using absolute NfL concentrations (*P* = 0.11) and a nonsignificant trend in NfL z-scores (*P* = 0.08, Fig. 3). Serum NfL concentrations (controlling for age and BMI) were also not correlated with total painDETECT scores for fibromyalgia (rho = 0.11, *P* = 0.44) or endometriosis (rho = 0.09, *P* = 0.54). Exploratory analyses of individual painDETECT items in fibromyalgia identified pain triggered by slight pressure as the most closely correlated with serum NfL levels, but this did not survive correction for multiple testing (NfL concentrations: r = 0.36, adjusted *P* = 0.09 [Supplemental Fig. 1, http://links.lww.com/PR9/A391], NfL z-scores: r = 0.11, adjusted *P* = 0.94 [Supplemental Fig. 2, http://links.lww.com/PR9/A391]).

## 4. Discussion

Participants with fibromyalgia and small fibre neuropathy had significantly elevated serum NfL levels compared with healthy controls, indicating similarities in serological signs of axonal injury. By contrast, participants with endometriosis demonstrated intermediate levels, with absolute NfL concentrations similar to controls but significantly elevated when comparing NfL z-scores. Using the painDETECT questionnaire, we identified neuropathic-like pain in 44% of fibromyalgia and 26% of endometriosis participants. For small fibre neuropathy, 96% met the criteria for probable or definite neuropathic pain according to NeuPSIG grading. Although we found no association between NfL levels and neuropathic-like pain presence and severity, there was a trend toward elevated NfL z-scores in participants with neuropathic compared with nonneuropathic pain. However, this comparison was limited by relatively small sample sizes and differences in clinical assessments and neuropathic pain classification tools across cohorts. Overall, our results contribute to growing evidence that a subset of participants with fibromyalgia have ongoing axonal injury and signs of neuropathic-like pain.

### 4.1. Fibromyalgia exhibits similar absolute serum neurofilament light chain concentrations to small fibre neuropathy

Our results show elevated serum NfL concentrations in fibromyalgia, similar to prior findings in a smaller cohort comparing plasma NfL levels in fibromyalgia with healthy controls.^42^ Although absolute NfL concentrations in fibromyalgia showed small but significant differences compared with controls, NfL z-scores revealed larger between-group differences. Despite these analytical variations, our findings add to well documented structural signs of small nerve fibre pathology in a subset of participants with fibromyalgia. Nearly 50% of participants with fibromyalgia exhibit signs of small nerve fibre pathology, with 45% (95% CI: 32%–59%) identified through skin biopsies and 59% (95% CI: 40%–78%) through corneal confocal microscopy.^29^ Functional nerve assessments using microneurography^48^ and quantitative sensory testing corroborate the structural pathology, demonstrating mixed sensory profiles with hypoaesthesia in a distinct subgroup of fibromyalgia.^18,41,56^ Taken together, these findings suggest that fibromyalgia is a heterogeneous condition with a subset of individuals displaying clear signs of ongoing structural and functional nerve pathology.

Unexpectedly, participants with fibromyalgia demonstrated comparable serum NfL concentrations to those with small fibre neuropathy, despite the latter representing our positive control for recognised peripheral neuropathy. This is particularly striking given that small fibre neuropathy is defined by peripheral nerve pathology determined by a combination of clinical signs, quantitative sensory testing, or skin biopsies,^15^ whereas such pathology is typically only present in a subset of participants with fibromyalgia. Elevated serum NfL concentrations in fibromyalgia and small fibre neuropathy is notable, suggesting ongoing axonal injury despite chronic symptoms (median duration >4 years). NfL is primarily released during active axonal injury, and its continued elevation indicates an ongoing degenerative process.^35,36^ This dynamic pathological process, continuing years after symptom onset, warrants further mechanistic characterisation.

Despite similar NfL concentrations, fibromyalgia and small fibre neuropathy have distinct clinical characteristics.^50^ Fibromyalgia typically presents with widespread pain and diffuse small fibre pathology, whereas small fibre neuropathy more often presents with distal pain and typically with length-dependent nerve fibre loss.^29,38,41,50^ These clinical distinctions suggest differing underlying mechanisms despite similar serological nerve pathology profiles.

### 4.2. Etiological considerations underlying elevated neurofilament light chain levels in fibromyalgia

Mechanisms contributing to nerve pathology in fibromyalgia are currently unknown and likely multifaceted.^10,46^ One hypothesis is that neurological sequalae may be derived from immune mechanisms.^17,40^ Notably, Goebel et al. demonstrated that IgG from fibromyalgia participants sensitizes nociceptive afferents in mice and induces loss of intraepidermal nerve fibre densities.^27^ Recent evidence extended these findings, showing that 37% (68/184) of sera from individuals with fibromyalgia bound to rat dorsal root ganglia, with FABP7 and TRPV1 binding positively correlating with current pain intensity and burning pain, respectively.^47^

Physical activity levels may also contribute to altered NfL levels, supporting the hypothesis that neuronal changes in fibromyalgia reflect downstream effects of activity reduction secondary to pain and fatigue. Although not allowing causal inferences, increased physical activity has been associated with reduced NfL concentrations in multiple sclerosis,^16,31^ dementia,^9^ and diabetes.^49^ A systematic review indicated that only 37% of people with fibromyalgia met recommended weekly activity guidelines.^57^ A pilot randomized controlled trial found that supervised physical activity increased intraepidermal nerve fibre densities in people with fibromyalgia at 18-month follow-up compared with those completing generic physical activity.^26^

Notably, NfL is not specific to peripheral axonal damage and may also reflect central changes. This is particularly relevant in fibromyalgia, as recent research has identified associations between serum NfL levels and depression,^51^ which is prevalent in fibromyalgia.^1^ An increased risk of developing dementia has also been linked to fibromyalgia, which may contribute to higher NfL levels.^54^ Our findings may reflect both central and peripheral involvement, potentially explaining the lack of association with neuropathic-like pain and emphasising fibromyalgia's heterogeneity. As the fibromyalgia and endometriosis cohorts lacked detailed peripheral nervous system assessments, the anatomical source of elevated NfL could not be determined. As NfL is expressed in both the central and peripheral nervous system, recent efforts have identified peripherin, another neuronal intermediate filament, as a nearly exclusive biomarker of peripheral nervous system injury.^5,34^ Future work using peripherally focussed biomarkers, such as peripherin in deeply phenotyped cohorts, may better localise nerve pathology in fibromyalgia.

### 4.3. Endometriosis demonstrates an intermediate neurofilament light chain phenotype

Endometriosis affects approximately 1 in 10 people assigned female at birth and is frequently associated with chronic pelvic pain. Although traditionally considered a nociceptive pain condition, recent work has identified subgroups with neuropathic-like pain.^11,13,14^ In our study, endometriosis represented an intermediate NfL phenotype, with similar absolute NfL concentrations compared with controls but elevated NfL z-scores. Given the lack of gold-standard analytical approaches for NfL data, we used both absolute concentrations (statistically controlling for age and BMI, compared with our control cohort) and individual age- and BMI-adjusted z-scores (derived from a previously published large international control cohort^6^). The elevated z-scores, despite similar absolute concentrations, may reflect differences in analytical approaches and control group characteristics. However, the intermediate NfL profile in endometriosis may also echo the lower prevalence of neural injury compared with other chronic pain conditions. Our findings of intermediate NfL levels underscore the need for further research into nerve involvement in endometriosis.

### 4.4. Neuropathic-like pain in traditionally non-neuropathic pain conditions

We observed varying levels of neuropathic-like pain across chronic pain cohorts, highlighting a continuum that likely reflects both differences in underlying pain mechanisms and assessment methods (painDETECT vs NeuPSIG grading). This was evident in fibromyalgia, where our exploratory correlation analysis suggested a positive association between serum NfL concentrations and pressure pain, although this did not survive correction for multiple testing. This aligns with previous studies in fibromyalgia showing similar associations between neuropathic pain questionnaire scores and numbers of painful pressure points,^43,55,58^ although questionnaires without neurological examination may not fully capture mechanistic pain phenotypes.^2^

Neuropathic-like pain across all 3 conditions reinforces the heterogeneity of pain mechanisms. Current assessment tools compound this complexity, as the painDETECT questionnaire has attracted criticism for identifying nociplastic pain components and correlations with signs of central sensitization.^7,25^ Objective markers of neural injury, such as NfL, provide quantifiable evidence of axonal injury. In fibromyalgia, elevated NfL levels and documented small fibre nerve pathology directly challenge its classification as purely nociplastic.^39^ Our findings support the view that nociplastic, neuropathic, and nociceptive mechanisms can coexist in varying proportions across individuals with chronic pain,^33^ supporting the need for personalized diagnostic and treatment approaches. Identifying signs of neuropathic-like pain and nerve pathology, including NfL, in fibromyalgia may improve patient stratification, enabling more targeted treatments and improved outcomes. Although we identified group differences in NfL levels, the high variability limits its value for individual patient assessment in clinical settings, and more sensitive markers are needed.

## 5. Limitations

Our study has limitations to consider. First, our retrospective multicohort design with limited phenotypic data introduces heterogeneity in pain phenotyping and the limited phenotypic data prevent the anatomical localisation of the identified nerve pathology. In the absence of data on neuropathic pain grading in the fibromyalgia and endometriosis cohorts, neuropathic-like pain was assessed using validated questionnaires, which, although commonly used, may underestimate the prevalence of neuropathic pain compared with clinical evaluations^30^ or misclassify nociplastic pain as neuropathic pain, including in fibromyalgia.^7,25^ Future studies should incorporate clinical assessments, following recommendations of the Neuropathic Pain Special Interest Group^19^; however, we acknowledge that this is challenging in conditions such as endometriosis. Second, there is currently no universally accepted method for analysing NfL levels. We carefully age-matched controls to the fibromyalgia group and adjusted for confounders including age and BMI. We further report both absolute concentration and z-score analyses based on a large population cohort. Although these results reveal medium-to-large effect sizes with large confidence intervals that differed for the endometriosis group between comparisons, elevated NfL in fibromyalgia and small fibre neuropathy remained consistent, strengthening the stability of our findings. Finally, we used serum samples from historical cohorts, which had slightly differing clinical measures and not all variables of interest were available in all cohorts, including skin biopsies. Future longitudinal studies with harmonized and more extensive phenotyping could provide additional information into the relationship between NfL levels and localization between peripheral and central nervous system pathology.

## 6. Conclusion

Our study provides serological signs of nerve pathology in a subset of participants with fibromyalgia, with elevated NfL levels comparable to small fibre neuropathy. Endometriosis represented an intermediate NfL phenotype. We identified neuropathic-like pain in a subset of participants with fibromyalgia, further challenging its traditional classification as a purely nociplastic pain condition. These findings suggest that fibromyalgia includes distinct pathophysiological subgroups and highlight the potential for NfL as a clinical biomarker to identify ongoing axonal injury in chronic pain.

## Disclosures

The authors have no conflict of interest to declare.

## Supplemental digital content

Supplemental digital content associated with this article can be found online at http://links.lww.com/PR9/A391.

## Supplementary Material

SUPPLEMENTARY MATERIAL
