# Supplementary material for: Serum neurofilament light chain in fibromyalgia: comparative evidence of neuronal injury across chronic pain conditions
Source: Pain Rep. 2026 Mar 24;11(2):e1423. doi: 10.1097/PR9.0000000000001423 (PMC13016184; doi:10.1097/PR9.0000000000001423)
Supplement: SUPPLEMENTARY MATERIAL [file painreports-11-e1423-s001.pdf]

Supplemental Material

<u>Supplementary Methods:</u>

Fibromyalgia

As part of the eligibility criteria for the randomised controlled trial, participants with fibromyalgia were also required to have self-reported difficulties with concentration or memory on the Daytime Functioning and Sleep Attribution Scale (DFSAS) (individuals who reported "*quite a bit*" or "*very much*" trouble on at least one of concentrating or focusing on things were eligible [5]) and reported insomnia, frequent waking, or early morning waking on the 2-item Sleep Condition Indicator (SCI-2) (individuals who reported problems sleeping at least 3 nights per week and who reported that poor sleep troubled them "much" or "very much" were eligible [4]). Participants with fibromyalgia were excluded if they had: a major neuropsychiatric condition (excluding depression and anxiety); diagnosis of primary neurological condition which could affect pain or cognitive assessment (e.g. Parkinson's disease, Dementia, cognitive impairment; Epilepsy); recent (<6 weeks) or planned surgery during trial period; current or planned nightshift work during trial period (>1 per week); untreated sleep disorder (e.g. obstructive sleep apnoea, restless leg syndrome, circadian rhythm disorder, parasomnia) [8]; taken prescribed sedative sleep medications on >2 nights in the past 2 weeks prior to study entry; received other psychological therapy for insomnia; currently pregnant or breastfeeding.

Endometriosis

In order to have sufficiently detailed pain-related data, we additionally selected participants from ENDOx who had also consented to a subsequent study (Translational Research in Pelvic Pain (TRiPP), n=23). TRiPP explored chronic pelvic pain more broadly, however only those in the endometriosis sub-groups of this study were included here. Ethical approval was given by the South Yorkshire Research Ethics Committee (19/YH/0030). Criteria for the endometriosis sub-groups required all participants to report at least one pelvic pain symptom of an intensity ≥ 4/10, have a confirmed surgical diagnosis of endometriosis and be aged 18-50 at the time of consent [2]. Most serum samples were collected on the day of endometriosis surgery following the WERF EPHect protocol [6].

Small Fibre Neuropathy

Diagnostic criteria for small fibre neuropathy included either: probable small fibre neuropathy: symptoms in hands and feet consistent with small fibre dysfunction (pain and altered temperature sensibility), clinical signs of small fibre damage (reduced pinprick sensitivity and ability to discriminate warm/cool), and normal nerve conduction studies; or definite small fibre neuropathy: symptoms in hands and feet, clinical signs of small fibre damage, normal nerve conduction studies, and altered intra-epidermal nerve fibre density at the ankle compared to normative reference values[1] and/or abnormal quantitative sensory testing of thermal thresholds at the foot. These criteria are derived from the diagnostic framework for small fibre neuropathy in diabetes mellitus[7] and closely align with the Devigili criteria for small fibre neuropathy. According to the Devigili criteria, a diagnosis requires the presence of at least two of the following three findings:

(1) clinical signs of small fibre damage, (2) abnormal thermal thresholds, or (3) reduced intra-epidermal nerve fibre density[3]. All participants with small fibre neuropathy satisfied the Devigili criteria. Intraepidermal nerve fibre data were available for 23/24 participants with small fibre neuropathy within our cohort.

Patients were excluded if they were pregnant, had coincident major psychiatric disorders, poor or no English language skills, documented central nervous system lesions, or insufficient mental capacity to provide informed consent or to complete clinical phenotyping.

Supplemental Table 1. Serum NfL levels

	Fibromyalgia (n=60)	Endometriosis (n=61)	Small fibre neuropathy (n=24)	Healthy control (n=30)
Serum NfL: pg/mL	8.52 (4.06)	5.57 (2.47)	8.98 (5.64)	6.77 (3.16)
NfL z-scores	-0.30 (1.18)	-0.41 (1.26)	0.23 (1.10)	-1.15 (0.86)

Data are expressed as mean (standard deviation). Abbreviations: NfL: neurofilament light chain

Supplemental Table 2. Serum NfL concentration (pg/mL) ANCOVA results

Contrast	Estimate	SE	T-ratio	P-value
healthy - fibromyalgia	-1.81	0.69	-2.61	**0.048**
healthy - endometriosis	-1.04	0.70	-1.50	0.44
healthy - SFN	-2.82	0.82	-3.46	**0.004**
fibromyalgia - endometriosis	0.76	0.61	1.25	0.60
fibromyalgia - SFN	-1.01	0.73	-1.40	0.50
endometriosis - SFN	-1.78	0.73	-2.42	0.08

Abbreviations: NfL: neurofilament light chain SE: standard error; SFN: small fibre neuropathy.

Supplemental Table 3. Serum NfL z-scores ANOVA results

Contrast	Estimate	SE	T-ratio	P-value
healthy - fibromyalgia	-0.85	0.26	-3.31	**0.006**
healthy - endometriosis	-0.74	0.26	-2.87	**0.024**
healthy - SFN	-1.38	0.32	-4.37	**0.0001**
fibromyalgia - endometriosis	0.12	0.21	0.55	0.95
fibromyalgia - SFN	-0.53	0.28	-1.89	0.23
endometriosis - SFN	-0.64	0.28	-2.31	0.10

Abbreviations: NfL: neurofilament light chain SE: standard error; SFN: small fibre neuropathy.

Supplemental Table 4. Adjusted Mean Differences and Effect Size Estimates of Serum NfL concentration (pg/mL) ANCOVA results

Contrast	Adjusted mean difference	p-value (Tukey)	Hedges' g	95% CI (g)
healthy – endometriosis	-1.04	0.439	-0.35	(-0.79, 0.09)
**healthy – fibromyalgia**	**-1.81**	**0.048**	**-0.61**	**(-1.05, -0.16)**
**healthy – SFN**	**-2.82**	**0.004**	**-0.94**	**(-1.50, -0.38)**
endometriosis – fibromyalgia	-0.76	0.599	-0.26	(-0.61, 0.10)
endometriosis – SFN	-1.78	0.077	-0.60	(-1.08, -0.12)
fibromyalgia – SFN	-1.01	0.503	-0.34	(-0.82, 0.14)

Bolded text indicates statistically significant adjusted mean differences (Tukey adjusted p-values < 0.05).
Abbreviations: CI: confidence interval; SFN: small fibre neuropathy.

Supplemental Table 5. Adjusted Mean Differences and Effect Size Estimates of Serum NfL z-scores ANOVA results

Contrast	Adjusted mean difference (z)	p-value (Tukey)	Hedges' g	95% CI (g)
**healthy – endometriosis**	**-0.74**	**0.024**	**-0.63**	**(-1.08, -0.19)**
**healthy – fibromyalgia**	**-0.85**	**0.006**	**-0.73**	**(-1.18, -0.28)**
**healthy – SFN**	**-1.38**	**0.0001**	**-1.18**	**(-1.76, -0.60)**
endometriosis – fibromyalgia	-0.12	0.946	-0.10	(-0.46, 0.26)
endometriosis – SFN	-0.64	0.099	-0.55	(-1.03, -0.07)
fibromyalgia – SFN	-0.53	0.235	-0.45	(-0.93, 0.02)

Bolded text indicates statistically significant adjusted mean differences (Tukey adjusted p-values < 0.05).
Abbreviations: CI: confidence interval; SFN: small fibre neuropathy.

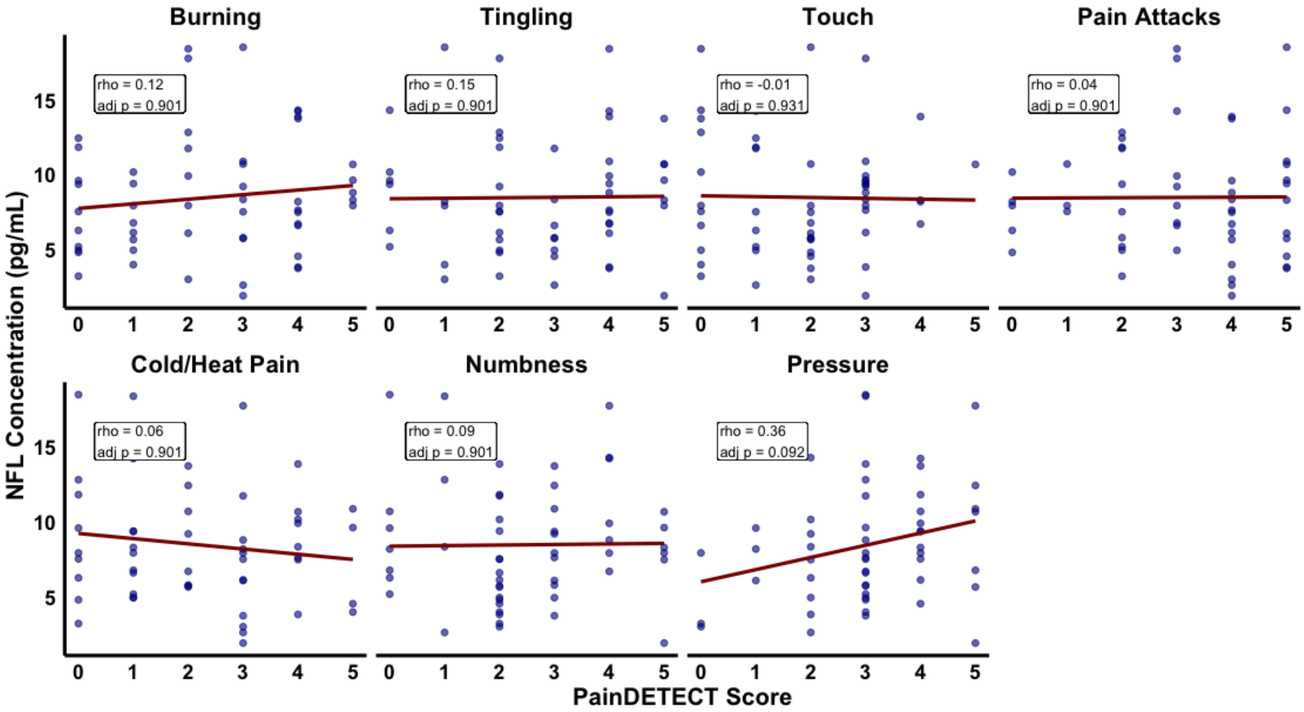

Supplemental Figure 1. Partial correlations for neuropathic pain characteristics using the painDETECT questionnaire and NfL concentrations in the fibromyalgia cohort. Partial correlations were adjusted for age and BMI.

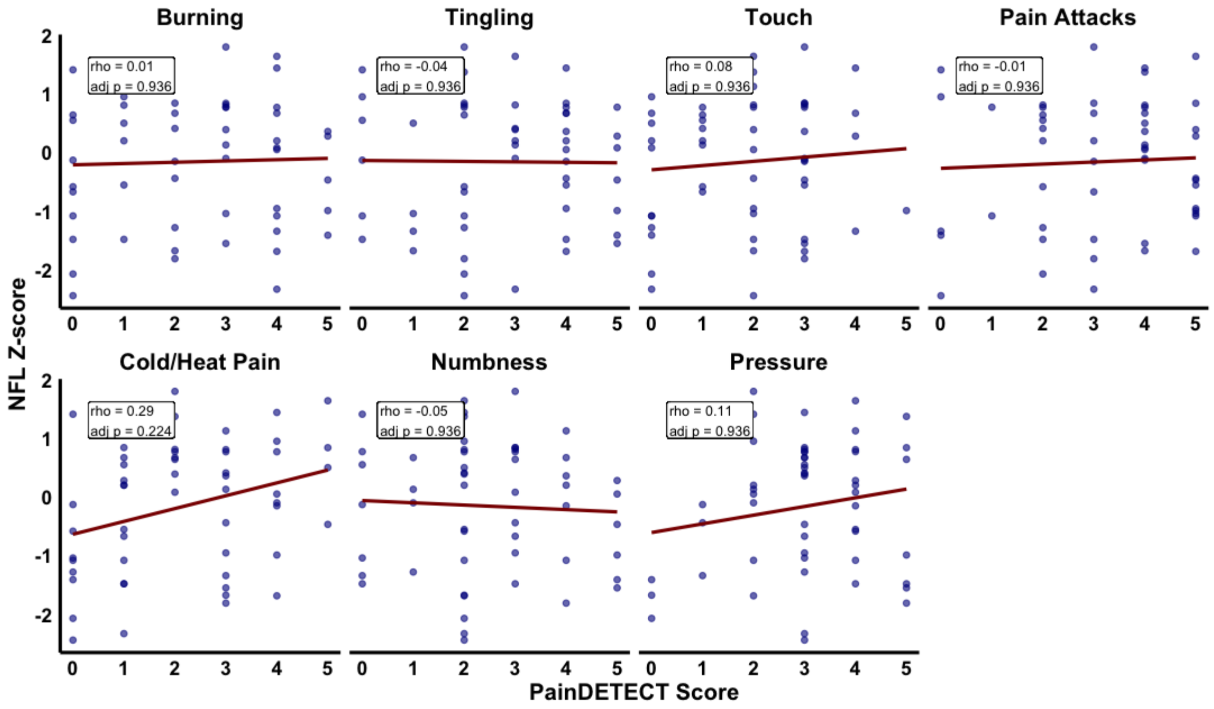

Supplemental Figure 2. Correlations for neuropathic pain characteristics using the painDETECT questionnaire and NfL z-scores in the fibromyalgia cohort.

References

[1] Bakkers M, Merkies ISJ, Lauria G, Devigili G, Penza P, Lombardi R, Hermans MCE, van Nes SI, De Baets M, Faber CG. Intraepidermal nerve fiber density and its application in sarcoidosis. Neurology 2009;73(14):1142-1148.

[2] Demetriou L, Coxon L, Krassowski M, Rahmioglu N, Arendt-Nielsen L, Aziz Q, Becker CM, Birch J, Cruz F, Hoffman A, Horne AW, Hummelshoj L, McMahon S, Meijlink J, Pogatzki-Zahn E, Sieberg CB, Tracey I, Treede R-D, Missmer SA, Zondervan KT, Nagel J, Vincent K. Deep phenotyping of women with endometriosis-associated pain and bladder pain syndrome: the TRiPP (Translational Research in Pelvic Pain) study protocol. medRxiv 2022:2022.2005.2016.22274828.

[3] Devigili G, Rinaldo S, Lombardi R, Cazzato D, Marchi M, Salvi E, Eleopra R, Lauria G. Diagnostic criteria for small fibre neuropathy in clinical practice and research. Brain 2019;142(12):3728-3736.

[4] Espie CA, Kyle SD, Hames P, Gardani M, Fleming L, Cape J. The Sleep Condition Indicator: a clinical screening tool to evaluate insomnia disorder. BMJ Open 2014;4(3):e004183.

[5] Kyle SD, Morgan K, Espie CA. THE DAYTIME FUNCTIONING AND SLEEP ATTRIBUTION SCALE (DFSAS): A NEW INSOMNIA-SPECIFIC MEASURE TO PROBE DAYTIME IMPAIRMENT AND POOR SLEEP ATTRIBUTIONS. Sleep 2010;33:A192-A193.

[6] Rahmioglu N, Fassbender A, Vitonis AF, Tworoger SS, Hummelshoj L, D'Hooghe TM, Adamson GD, Giudice LC, Becker CM, Zondervan KT, Missmer SA, Adamson GD, Allaire C, Anchan R, Becker CM, Bedaiwy MA, Buck Louis GM, Calhaz-Jorge C, Chwalisz K, D'Hooghe TM, Fassbender A, Faustmann T, Fazleabas AT, Flores I, Forman A, Fraser I, Giudice LC, Gotte M, Gregersen P, Guo SW, Harada T, Hartwell D, Horne AW, Hull ML, Hummelshoj L, Ibrahim MG, Kiesel L, Laufer MR, Machens K, Mechsner S, Missmer SA, Montgomery GW, Nap A, Nyegaard M, Osteen KG, Petta CA, Rahmioglu N, Renner SP, Riedlinger J, Roehrich S, Rogers PA, Rombauts L, Salumets A, Saridogan E, Seckin T, Stratton P, Sharpe-Timms KL, Tworoger S, Vigano P, Vincent K, Vitonis AF, Wienhues-Thelen UH, Yeung PP, Jr., Yong P, Zondervan KT. World Endometriosis Research Foundation Endometriosis Phenome and Biobanking Harmonization Project: III. Fluid biospecimen collection, processing, and storage in endometriosis research. Fertility and Sterility 2014;102(5):1233-1243.

[7] Tesfaye S, Boulton AJM, Dyck PJ, Freeman R, Horowitz M, Kempler P, Lauria G, Malik RA, Spallone V, Vinik A, Bernardi L, Valensi P, Group obotTDNE. Diabetic Neuropathies: Update on Definitions, Diagnostic Criteria, Estimation of Severity, and Treatments. Diabetes Care 2010;33(10):2285-2293.

[8] Wilson SJ, Nutt DJ, Alford C, Argyropoulos SV, Baldwin DS, Bateson AN, Britton TC, Crowe C, Dijk DJ, Espie CA, Gringras P, Hajak G, Idzikowski C, Krystal AD, Nash JR, Selsick H, Sharpley AL, Wade AG. British Association for Psychopharmacology consensus statement on evidence-based treatment of insomnia, parasomnias and circadian rhythm disorders. J Psychopharmacol 2010;24(11):1577-1601.
